# ﻿A realigned taxonomy for the *Schiedeakauaiensis* – *S.perlmanii* species pair (Caryophyllaceae) based on recent collections and new analyses that require nomenclatural changes for both species

**DOI:** 10.3897/phytokeys.254.148438

**Published:** 2025-03-26

**Authors:** Warren L. Wagner, Stephen G. Weller

**Affiliations:** 1 Department of Botany, MRC-166, National Museum of Natural History, Smithsonian Institution, P.O. Box 37012, Washington, DC 20013-7012, USA National Museum of Natural History, Smithsonian Institution Washington United States of America; 2 Department of Ecology and Evolutionary Biology, University of California, Irvine, CA 92697, USA University of California Irvine United States of America

**Keywords:** Caryophyllaceae, Conservation, Hawaiian Islands, Kaua‘i, Schiedea

## Abstract

The Kaua‘i species *Schiedeakauaiensis* H. St. John was previously characterized by a geographical range including a number of Napali Coast valleys and the Limahuli, Wainiha, and Manoa valleys in the northern part of the island whereas the closely related *S.perlmanii* W. L. Wagner & Weller occurs in the Anahola area and on Ha‘upu on the windward (eastern) side of Kaua‘i. The primary characteristic distinguishing them is a subshrub habit for *S.kauaiensis* vs a vining habit in *S.perlmanii*. In several localities from northern Kaua‘i including Limahuli, Wainiha, and Manoa valleys, populations were known only from herbarium specimens but were included within *S.kauaiensis* in part because these localities were closest to the Napali Coast valleys, which encompasses the remainder of the range of the species. Recent field work resulting in discovery of new populations and cultivation of plants from Limahuli and Manoa has shown that plants from these three northern localities do not represent *S.kauaiensis* but rather fit with *S.perlmanii*. Two of the collections from this northern area are the types of *S.wichmanii* H. St. John and *S.kauaiensis*. Since these names were published earlier, we must adopt here the earliest name, *S.kauaiensis*, for the plants formerly known as *S.perlmanii* leaving the species from the Napali Coast valleys without a name and described here as a new species, *S.napaliensis* W. L. Wagner & Weller.

## ﻿Introduction and discussion

The Kaua‘i Island pair of closely related species of Schiedea Cham. & Schltdl. in sect. Mononeura W.L. Wagner & Weller, *S.kauaiensis* and *S.perlmanii* were distinguished by growth habit and geography in the most recent revision of the genus (Wagner et al. 2005), where *S.perlmanii* was first published. In that publication, *Schiedeakauaiensis*, a subshrub, was noted as occurring on the northwestern to northern part of the island and *S.perlmanii* had a vining habit and occurred from the southeastern to northeastern part of the island. In general, *S.perlmanii* occurred in lower elevation mesic vegetation and was known from only a few collections from the Ha‘upu area of southeastern Kaua‘i and from a single collection in 1952 disjunct from Ha‘upu in the Anahola area above Papa‘a in northeastern Kaua‘i. *Schiedeakauaiensis* was known from scattered areas of Kaua‘i, including Wainiha-Manoa ridge (type locality), Limahuli, Kalalau, Nualolo, and Mahanaloa valleys on the northern to western side, and Olokele Valley and the Wahiawa Mountains in the central region, in open areas of diverse mesic forest at a wider range of elevations. However, there were only three herbarium collections available for study from the northern areas of Limahuli, Wainiha, and Manoa valleys and we had no direct field observations or cultivated plants in the greenhouse for study. Since these localities were close to the eastern end of the range of *S.kauaiensis* and the primary distinguishing character (habit) was not clear on herbarium specimens, they were included in the delimitation of *S.kauaiensis*. After publication of the *Schiedea* monograph in 2005, new collecting activity on the eastern and northern areas of Kaua‘i, especially from 2014, and 2021–2023, led to the rediscovery of populations of *S.perlmanii* on Kawalumakua (near Papa’a) and of *S.kauaiensis* in Manoa and Limahuli valleys. Collectors (Heintzman and Wood) noted that the collections from Manoa were morphologically atypical and very close to *S.perlmanii*.

Recent comprehensive sampling across the genus used a variety of greenhouse, field and laboratory studies to gain additional insights into the evolution of breeding systems in *Schiedea*. The phylogenetic focus of the project employed a suite of modern DNA sequencing tools to generate trees of hypothesized evolutionary relationships for all species in the genus. This was coupled with additional field and greenhouse studies to explore breeding system evolution, ranging from chemical analyses of floral scent to evening field observations of *Schiedea* flowers in the wild to better understand whether organisms such as moths might act as pollinators. The project also used progeny from crosses among the few remaining individuals of *S.kauaiensis* from several Napali Coast valleys for outplanting in natural areas. This focus allowed us to examine more closely the morphology of many species, including *S.kauaiensis* and *S.perlmanii*. A preliminary analysis of genomic data from ca. 25 samples of all known localities of these two species strongly supports the inclusion of populations from Limahuli and Manoa with *S.perlmanii* rather than with populations of *S.kauaiensis* from the Napali Coast (McDonnell et al., pers. comm.). With support from both morphological and genomic data we here move these three populations for inclusion in a recircumscribed species consisting of largely windward populations and resulting in a more narrowly circumscribed species on the NW leeward Napali valleys.

## ﻿Realigned taxonomy

We here utilize the information published previously in the *Schiedea* monograph (Wagner et al. 2005) with various updates to support the realigned classification with the removal of populations from Limahuli, Wainiha, and Manoa valleys on the northern part of Kaua‘i from the circumscription of what is described here as a new species, *S.napaliensis*. Because the type of *S.kauaiensis* was from Wainiha Valley, populations from Moloa‘a and Ha‘upu, which were formerly placed in *S.perlmanii*, will in the revised circumscription bear the oldest name *S.kauaiensis*, with *S.perlmanii* becoming a synonym of *S.kauaiensis*.

Following the new alignment of populations, the next step was to reexamine the morphological characters of all collections from throughout the ranges of both species. In addition to inflorescence size and habit, variation in flower size and pubescence in particular differentiated *S.kauaiensis* and *S.napaliensis*. A summary of most useful characters for distinguishing the two species is presented in Table 1.

**Table 1.** Comparison of morphological and geographical/ecological characters of the subclade of species of *Schiedeakauaiensis* and *S.napaliensis* (sect. Mononeura).

Character	*S.kauaiensis*	*S.napaliensis*
Habit	Vine, stems 6–12 dm long (in cultivation eventually to 15+ dm long), sprawling when young to reclining when longer, at least sparingly branched, glabrous throughout or sparsely short-puberulent in inflorescence	Erect to ascending subshrubs 3–10 dm tall, few branched, glabrous throughout, except glandular-puberulent throughout inflorescence
Leaf shape	Narrowly ovate or lanceolate to elliptic-lanceolate	Oblong-elliptic
Leaf length/width	Blades 4–11.5 cm long, 2–2.8 cm wide	Blades 7.5–15 cm long, 1.8–4.1 cm wide
Inflorescence	Inflorescence terminal, with 40–60 flowers, 20–35 cm long and nearly as wide, laterally-directed or pendent, the tertiary and higher level internodes or pedicels weakly spreading	Inflorescence terminal, with 27–70 flowers, 20–48 cm long, branches spreading
Bracts	Bracts subulate, the lowermost of central axis narrowly elliptic, falcate, green and purple-tinged or purple, the lowermost ones 2–17 mm long, those of branches and flowers 1.5–4.5 mm long, purple, the adaxial surface puberulent	Bracts subulate, the lowermost of the central axis elliptic-lanceolate, recurved and often twisted, as green as the leaves, the lower ones 30–45 mm long, those of the branches and flowers 5–18 mm long, glandular-puberulent
Pedicels	Pedicels (5–)13–15 mm long	Pedicels (7–) 10–23 mm long
Sepals	Sepals 2.2–3 mm long, ovate, green, sometimes purple-tinged toward apex or irregularly purple throughout, opaque, strongly reflexed, apex attenuate	Sepals 4.3–4.8 mm long, lanceolate, green, opaque, strongly reflexed, apex long-attenuate
Capsules	Capsules 2.5–2.8 mm long, ovoid	Capsules 3.1–3.8 mm long, narrowly ovoid
Seeds	Seeds ca. 1.2 mm long	Seeds ca. 1.3 mm long
Elevation/ habitat	400–640 m Mesic shrubland to mesic forest	750–950 m Diverse mesic forest

### ﻿ Schiedea kauaiensis

Taxon classificationPlantaeCaryophyllalesCaryophyllaceae

H. St. John, Phytologia 64: 177. 1988.

45EF2FDD-6ED5-5E81-B841-D55735648E6A

Figs 1 , 2 , 3

Schiedea nuttallii var. pauciflora O. Deg. & Sherff in Sherff, Bot. Leafl. 7: 6. 1952.Type. Hawaiian Islands, Kaua‘i: Forest Reserve, ridge behind Papa‘a, 16 Jan 1952, *O. Degener & A. B. Greenwell s .n.* (holotype: F-1451309, F-1451310, originally mounted on a single sheet [photo: F!], but now on 2 sheets [photos: F!]; isotypes: B! BISH-2 sheets!, F!, K, NY-2 sheets!, PH-2 sheets!, US- 2 sheets!).
Schiedea nuttallii var. lihuensis Sherff, Bot. Leafl. 9: 3. 1954. Type. Hawaiian Islands, Kaua‘i: [southeastern Kaua’i] “mauka of Gap?, near Lihue,” 1911, *J. M. Lydgate s. n.* (holotype: BISH-501710!, photo: F!, isotypes: BISH-2 sheets!)
Schiedea wichmanii H. St. John, Phytologia 64: 178. 1988. Type. Hawaiian Islands, Kaua‘i: Limahuli Valley, E wall, 60° slope, locality on dark soil and loose rock with remnant ‘Ohi‘a, Eugenia, Uluhe, Santalum [*pyrularium* A. Gray], Diospyros, Psychotria, and Hibiscus, 1300 ft [395 m], 13 Sep 1978, *S. Perlman & C. Wichman, Jr. 219* (holotype: BISH-522858!; isotype: BISH!, PTBG!). [Sterile specimens, flowers liquid preserved.]
Schiedea perlmanii W. L. Wagner & Weller, Syst. Bot Monogr. 72: 71. 2005. Type. Hawaiian Islands, Kaua‘i: Mt. Ha‘upu, near Queen Victoria’s profile, W of head of Victoria, *Diospyros-Metrosideros* lowland mesic forest, 1700–1950 ft [515–590] m, 27 Feb 1992, *S. Perlman 12614* (holotype: US-3252201!; isotypes: AD, BISH!, PTBG!).

#### Type.

**Hawaiian Islands, Kaua‘i** • Wainiha-Manoa ridge, wet forest near edge of pali, 2000 ft [610 m], 30 Jul 1977, *C. Christensen 290* (holotype: BISH-522854!).

**Figure 1.**
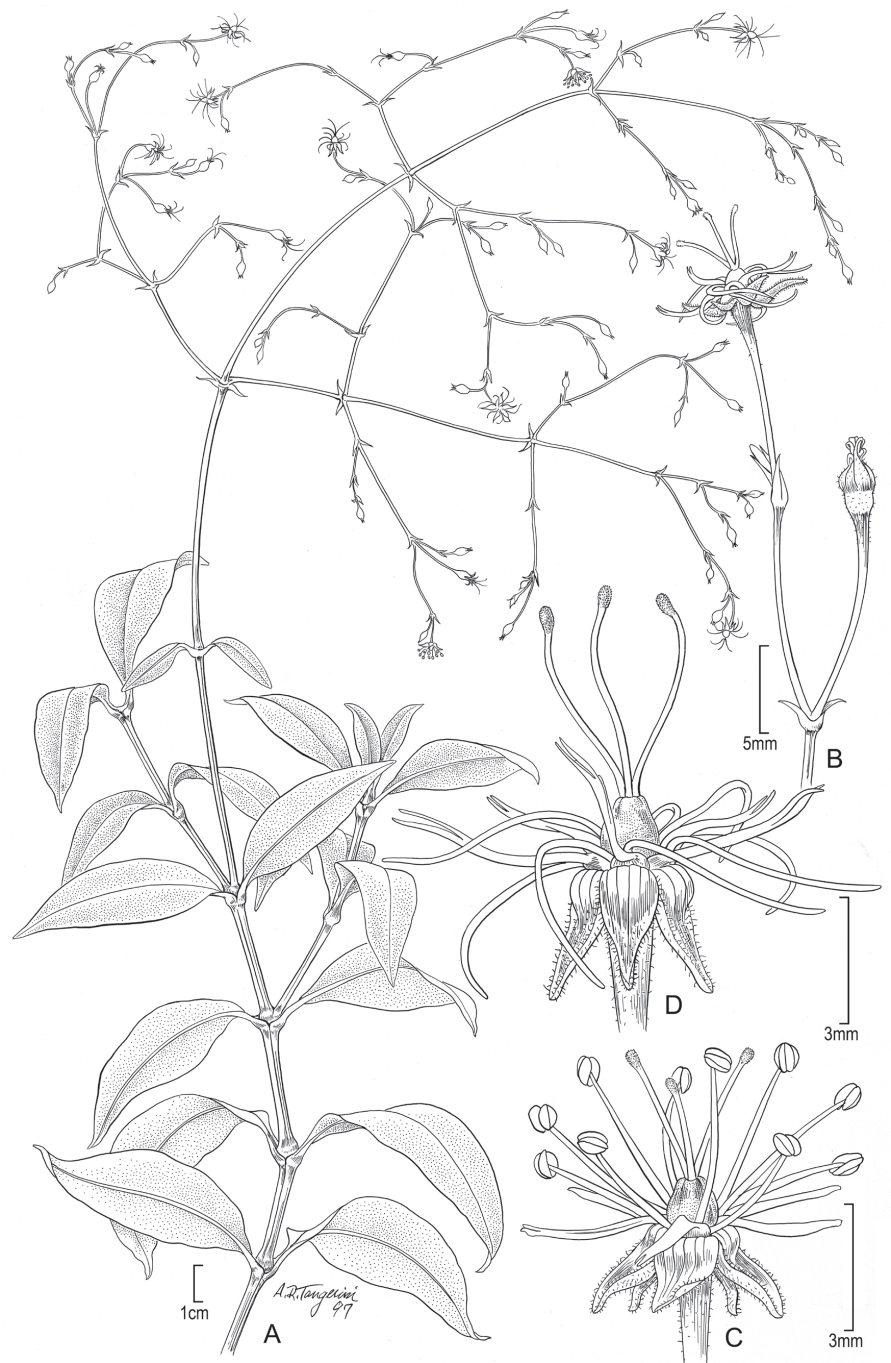
*Schiedeakauaiensis***A** habit, stem with leaves and inflorescence **B** branch of inflorescence **C** flower in early anthesis, male stage **D** flower in later anthesis, female stage. Reproduced from fig. 25 of Wagner et al. 2005; drawn from cultivated plant from *Perlman 12917* grown in the greenhouse of the University of California at Irvine. Illustration by Alice Tangerini.

#### Description.

Vine; stems 6–12 dm long (in cultivation eventually to 15 dm or more long), sprawling when young to reclining when longer, at least sparingly branched, internodes deep purple or purplish green, glabrous throughout, except bracts, sepals, and sometimes the pedicel sparsely short-puberulent. Leaves opposite; blades 4–11.5 cm long, 2–2.8 cm wide, narrowly ovate or lanceolate to elliptic-lanceolate, weakly glossy, green or yellowish green, sometimes purple-tinged, especially on lower surface, weakly coriaceous and rubbery, chartaceous when dry, with only the midvein evident, the midvein ± slightly excentric, usually reddish purple, margin entire, slightly thickened and becoming revolute toward the base, apex acute to acuminate; petioles 0.8–0.9 cm long, purple, weakly ± grooved. Inflorescence terminal, with 40–60 flowers, 20–35 cm long and nearly as wide, diffuse, laterally-directed or pendent, the tertiary and higher level internodes or pedicels weakly spreading; bracts subulate, the lowermost of central axis narrowly elliptic, falcate, green and purple-tinged or purple, the lowermost ones 2–17 mm long, those of branches and flowers 1.5–4.5 mm long, purple, the adaxial surface puberulent; pedicels 13–15 mm long at anthesis, weakly flattened, very weakly angled just below the flower and often sparsely short-puberulent. Flowers hermaphroditic, usually pendent. Sepals 2.2–3 mm long, elongating to 4 mm long in fruit, ovate, green, sometimes purple-tinged toward apex or irregularly purple throughout, opaque, strongly reflexed and convex in the proximal 1/4, producing a conspicuous transverse bulge, the distal part broadly navicular, oriented at 5° to 30° angle to the pedicel, abaxial side sparsely puberulent toward the base, the adaxial side puberulent, primarily near the midrib, margins conspicuously scarious, ciliate, apex attenuate, inconspicuously slightly twisted. Nectary base 0.7–0.9 mm long, yellow, the nectary shaft 4.5 mm long, gently recurved, at 90° angle to the axis, apex deeply bifid to ca. 1/2 their length. Stamens 10; filaments dimorphic, the antisepalous whorl 6.2–6.3 mm long, the alternate whorl 5 mm long; anthers 0.75–0.8 mm long, subequal, pale yellow. Styles 3. Capsules 2.5–2.8 mm long, ovoid. Seeds ca. 1.2 mm long, orbicular-reniform, compressed, the surface rugose. Chromosome number unknown.

**Figure 2.**
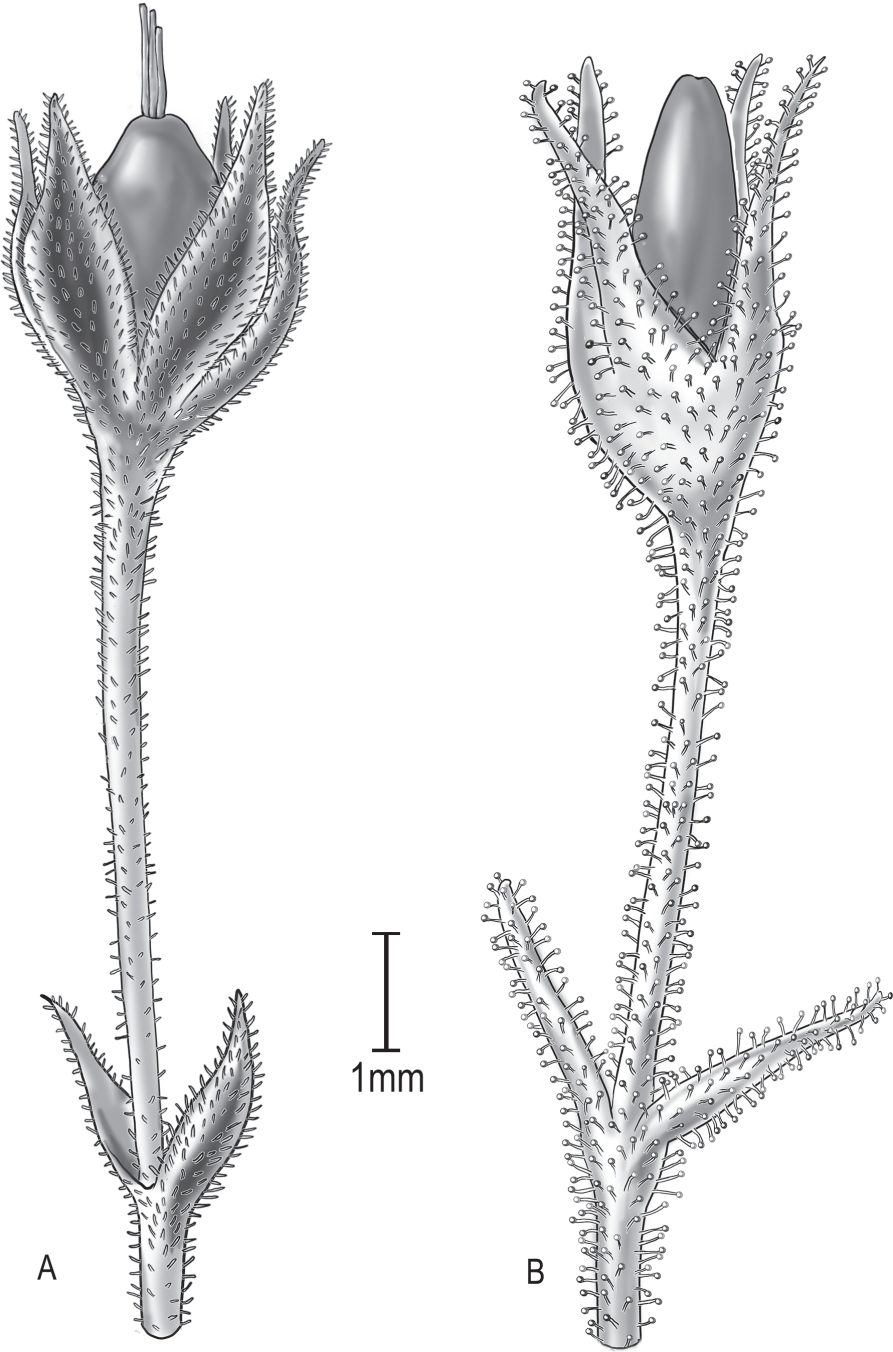
Comparison of inflorescence pubescence **A***Schiedeakauaiensis*, showing sparse short hairs **B***S.napaliensis*, glandular-puberulent throughout. Although seeds of *S.kauaiensis* and *S.napaliensis* are similar in size, mature capsules of *S.napaliensis* are larger and contain more seeds than *S.kauaiensis*. Drawn from herbarium specimen of *Perlman 17563* (US) and *Perlman 472* (**A**), and *Perlman 12074* (US) (**B**). Illustration by Alice Tangerini.

#### Distribution.

**Figure 3.**
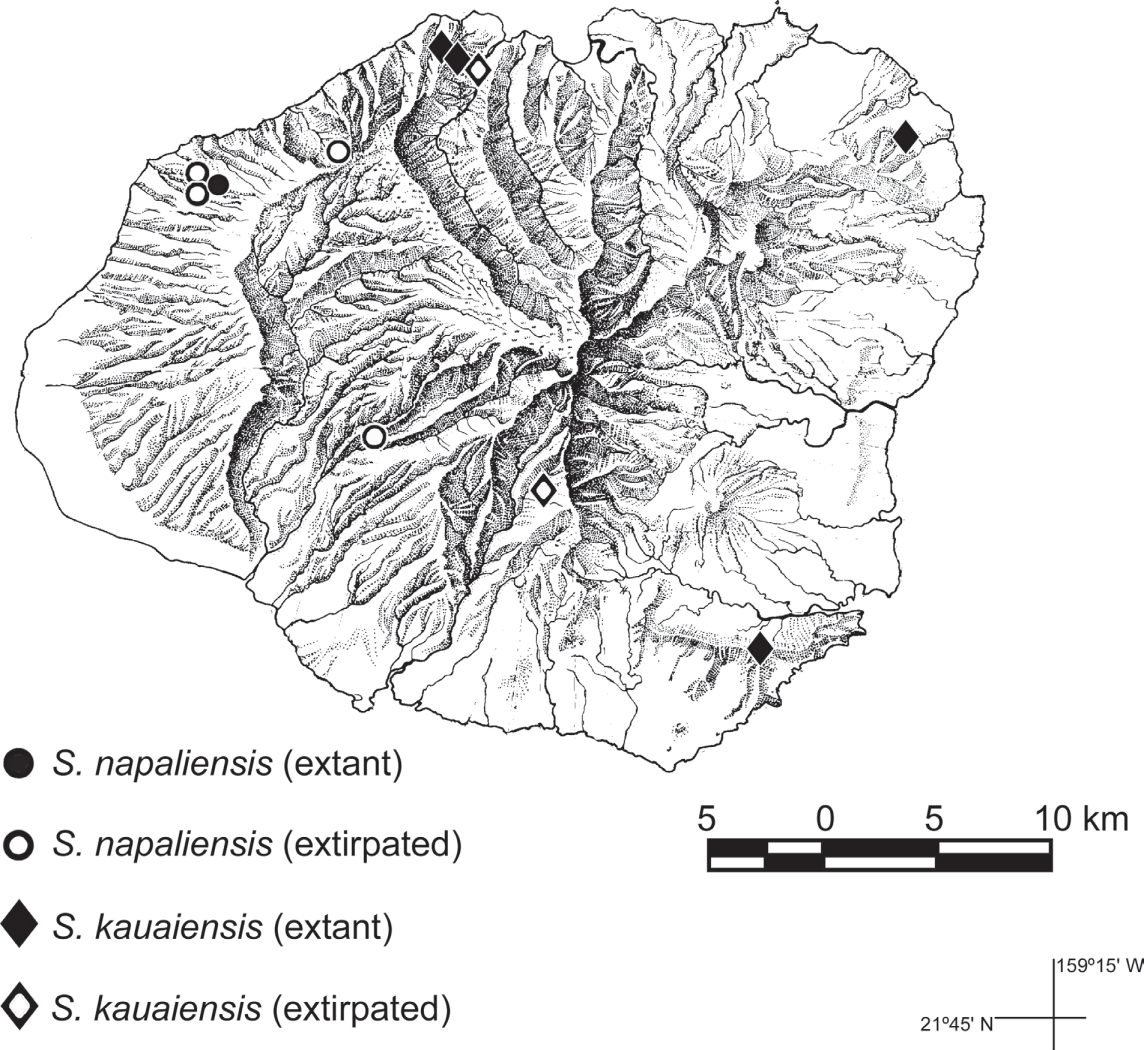
Distribution map (Kaua‘i, Hawaiian Islands) of *Schiedeakauaiensis* and *S.napaliensis* showing extant and extirpated locations as of 2024 for populations represented by collections. Reproduced from Fig. 26 of Wagner et al. (2005).

(Fig. 3). Kaua‘i, known from mesic shrubland in four disjunct areas on windward Kaua‘i: 1) near the summit of the Hoary Head Mountains (Ha‘upu), 2) from a collection from above Papa‘a made over 50 years ago and more recently Moloa‘a Forest Reserve, Anahola, 3) as well as Wainiha, Limahuli, and Manoa valleys, and 4) from an old collection of a population in Wahiawa Mountains (Hi‘i Mts) in the central region; ca. 400–640 m.

#### Specimens examined.

**Hawaiian Islands. Kaua‘i**: Koloa District: Hi‘i Mts., *s.d.*, *Lydgate s.n.* (BISH). Lihue District: Ha‘upu summit, slopes of N facing side near top, [21°55'33.6"N, 159°24'1.9"W], *Perlman et. al. 12917* (PTBG), *Wood et al. 18289* (PTBG), *Wood et al. 18449* (PTBG); Mt. Ha‘upu, N facing cliffs above Kipu, between Queen Victoria’s Profile and Mt. Ha‘upu summit, *Perlman 17563* (BISH, NY, PTBG, US), *Wood et al. 11435* (PTBG); Mt. Ha‘upu, slopes above Kipu ranch, to W of Queen Victoria’s Profile, *Perlman 17439* (PTBG), *17449* (PTBG); windward Ha‘upu, just E of Ha‘upu Peak, Kipu, below “Queen Elizabeth’s [Queen Victoria’s] Profile,” *Warshauer 1184* (BISH). Kawaihau District: Moloa‘a Forest Reserve, near Kawalumakua Peak [22°09'14.4"N, 159°20'08.4"W], *Wood et al. 14705* (PTBG), [22°09'19.9"N, 159°20'31.1"W], *Wood et al. 14718* (BISH, PTBG, US), *Wood et al. 14719* (PTBG), *Tangalin 3361* (PTBG). Hanalei District: Manoa valley, above falls in valley to East of Limahuli, 530 m, *Perlman et al. 23977* (PTBG); Wainiha valley, on ridge 1300 ft. S of Kulanaililia, top of ridge, [22°12'20.5"N, 159°34'5.1"W], *Christensen 317* (BISH); Limahuli Valley, E wall, 60° slope, locality on dark soil and loose rock with ‘Ohi‘a, *Eugenia*, Uluhe, *Santalum* [*pyrularium* A. Gray], *Diospyros*, *Psychotria*, and *Hibiscus*, 1300 ft [395 m], 16 August 1978, *S. Perlman & C. Wichman, Jr. 218* (BISH); Lower Limahuli valley, up subgulch on W side of valley [22°13'02.9"N, 159°34'59.6"W], *Perlman & Bender 17370* (PTBG).

#### Cultivated specimens.

**Kaua‘i**: Ha‘upu summit, slopes of N facing side near top, *Perlman et.al. 12917* [cult. *Wagner & Shannon 6795*] (BISH, GH, NY, PTBG, US); SE portion of Moloa‘a Forest Reserve, Anahola upper gulch [22°’15"N, 159°33"W], *Heintzman KP06012199* (US); from a cutting from Upper Manoa Valley, *Heintzman KP05052302* (PTBG), *Wood & DeMotta 18282* (PTBG).

### ﻿ Schiedea napaliensis

Taxon classificationPlantaeCaryophyllalesCaryophyllaceae

W.L.Wagner & Weller sp. nov.

1F09B221-1876-5171-AAF8-8AD3FD09466B

urn:lsid:ipni.org:names:77359323-1

Figs 2 , 3 , 4

#### Type.

**Hawaiian Islands, Kaua‘i** • Mahanaloa Valley, up valley from old horse trail, S side of valley, [22°12'35.6"N, 159°34'27.2"W], 10 July 1991, *S. Perlman & J. Obata 12074* (holotype: PTBG-1000046146!; isotypes: BISH, MO, US!).

**Figure 4.**
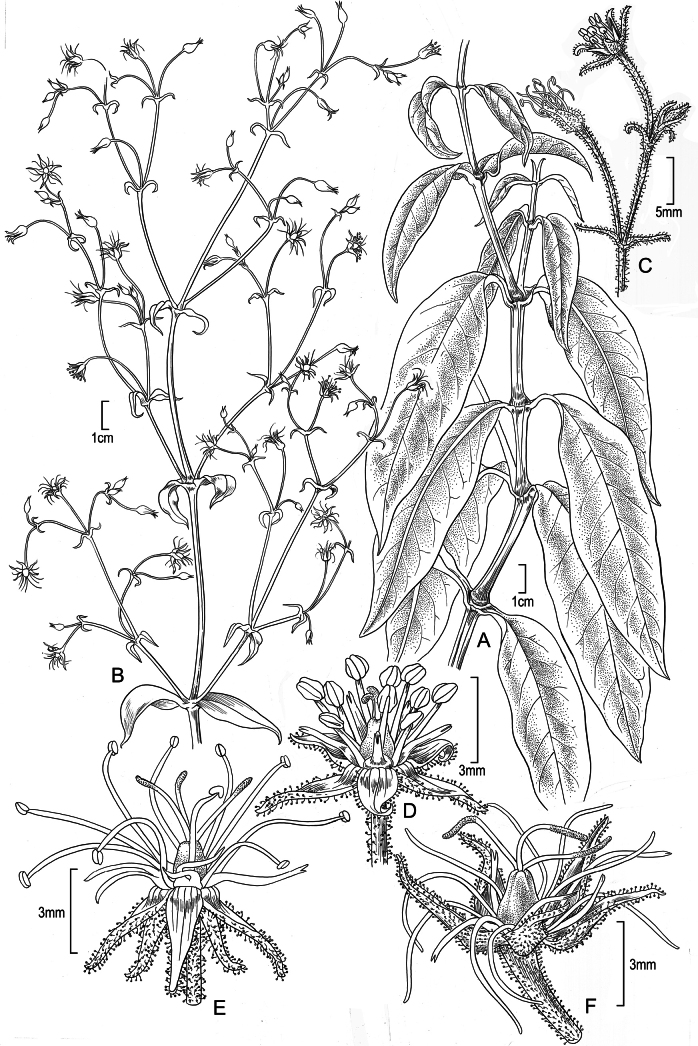
*Schiedeanapaliensis***A** habit, stem with leaves **B** inflorescence **C** portion of inflorescence **D** flower in early anthesis, male stage **E** flower in later anthesis, male stage **F** flower in female stage; Reproduced from fig. 27 of Wagner et al. 2005; drawn from cultivated plant from *Perlman 12074* grown in the greenhouse of the University of California at Irvine. Illustration by Alice Tangerini.

#### Description.

Erect to ascending subshrubs 3–10 dm tall; stems few-branched, glabrous, becoming sparsely then moderately glandular-puberulent in the inflorescence, the internodes purple. Leaves opposite; blades 7.5–15 cm long, 1.8–4.1 cm wide, oblong-elliptic, light green or yellowish green, adaxial surface slightly glossy, the abaxial surface glossy, slightly thickened and rubbery, chartaceous when dry, usually slightly undulate, with only the midvein evident or sometimes with an additional pair of inconspicuous, smaller, looping veins, the midvein ± slightly excentric, margin entire, slightly thickened and weakly revolute, especially toward the base, apex acute to weakly acuminate, base gradually attenuate; petioles 0.5–1.1 cm long, pale green, purple toward the base, weakly ± grooved. Inflorescence terminal, with 27–70 flowers, 20–48 cm long, diffuse, flowers widely spaced, branches spreading, progressively more densely puberulent to apex, the hairs straight, erect, 0.1–0.35 mm long; bracts subulate, the lowermost of the central axis elliptic-lanceolate, as green as the leaves, recurved and often twisted, the lower ones 30–45 mm long, those of the branches and flowers 5–18 mm long; pedicels (7–) 10–23 mm long, elongating slightly in fruit, slightly asymmetrically flattened. Flowers hermaphroditic. Sepals 4.3–4.8 mm long, lanceolate, green, opaque, strongly reflexed and convex in the proximal 1/4, producing a small transverse bulge, the distal part shallowly concave, oriented ca. 40° to 80° angle to the pedicel, glandular-puberulent, a few of the hairs sometimes non-glandular, margins scarious, ciliate, apex long-attenuate. Nectary base 0.6–0.9 mm long, dark yellow, the nectary shaft 3–4.5 mm long, gently recurved, at 90° to the axis, apex deeply bifid, sometimes divided nearly to the base. Stamens 10; filaments dimorphic, the antisepalous whorl 7.5 mm long, the alternate whorl 5.3–5.8 mm long; anthers 0.75–0.85 mm long, subequal, pale yellow. Styles 3. Capsules 3.1–3.8 mm long, narrowly ovoid. Seeds ca. 1.3 mm long, orbicular-reniform, compressed, the surface rugose. Chromosome number unknown.

#### Etymology.

Specific epithet refers to the geographic region of the Napali Coast valleys where this species occurs.

#### Specimens examined.

**Hawaiian Islands. Kaua‘i**: Waimea District: Olokele Valley, *Lydgate 12* (BM-BM013854574); Kopiwai, Ku‘ia Valley, [22°08'9.6"N, 159°41'32.7"W], *Hobdy 200* (BISH, US); Ku‘ia Natural Area Reserve, in Mahanaloa Valley, N facing slope of valley N of Milolii Ridge, above confluence with Pa‘aiki Valley, [22°08'1.4"N, 159°41'48.5"W], *Lorence & Wood 7620* (BISH [2], MO, PTBG); Mahanaloa Valley, above confluence of Kuia & Mahanaloa stream, 756 m, *Wood 7430* (PTBG, US); Mahanaloa Valley, below confluence of Kuia & Mahanaloa stream, 700 m, *Tangalin & Demotta 1981* (PTBG); Mahanaloa Valley, East from Weller #2, 701 m, *Tangalin & Aguraiuja 1953* (PTBG); Ku‘ia Valley, a tributary of Mahanaloa Valley, 200 ft inside Ku‘ia, right side slope, [22°8'17.2"N, 159°42'3"W], *Perlman 472* (BISH, US); Makaha Valley, 823 m, *Wood et al. 15662* (PTBG), 790 m, *Wood & Perlman 17429* (PTBG); Makaha valley, near bottom of gulch, North facing slope, 772 m, *Perlman et al. 25234* (PTBG, US); Nuololo, north facing slopes above drainage, 954 m, *Wood & Query 14517* (PTBG), *Wood et al. 15266* (BISH, PTBG, US), *Wood et al. 15568* (BISH, PTBG, US), *Wood et al. 15670.01* (PTBG). Hanalei District, Kalalau Valley, in back of valley, native cliffs and ridges, along ridge, [22°09'7.2"N, 159°37'42.8"W], *Wood et. al. 1973* (PTBG, US).

#### Cultivated specimens.

Kaua‘i. Mahanaloa Valley, up valley from old horse trail, S side of valley, *Perlman & Obata 12074* [cult. *Wagner & Shannon 6805*] (BISH, PTBG, US), *Perlman & Obata 12074* [cult. 1991, *Weller & Sakai s.n.*] (US).

#### Distribution.

(Fig. 3). *Schiedeanapaliensis* occurs in Waimea and Hanalei districts in the Napali Coast valleys of Makaha, Nualolo, Mahanaloa, Ku’ia, Pa’aiki, and Kalalau, and formerly in the Olokele Valley in the Waimea District, in open areas of diverse mesic forest; 750–950 m.

##### ﻿Conservation status

Only a single naturally established plant of *S.napaliensis* occurs in the wild at present. The causes for the decline of this species include browsing by introduced ungulates, erosion resulting from ungulate activity, and consumption of seedlings by introduced mollusks. Using seed collections or plants propagated in tissue culture at the Lyon Arboretum, plants representing three localities (Nualolo, Mahanaloa, and Makaha) were used in a greenhouse crossing program to produce outcrossed seeds for restoration efforts. Numerous plants have been introduced into protected areas on Kaua‘i by the Plant Extinction Prevention Program, Division of Forestry and Wildlife, State of Hawai‘i, and appear to be growing well. Whether these plants will produce seeds capable of establishing new generations of plants remains to be seen.

## Supplementary Material

XML Treatment for Schiedea kauaiensis

XML Treatment for Schiedea napaliensis

## ﻿Additional information

### Author ORCIDs

Warren L. Wagner https://orcid.org/0000-0001-5012-8422

### Data availability

All of the data that support the findings of this study are available in the main text.
